# Variations between post- and pre-harvest seasons in stunting, wasting, and Infant and Young Child Feeding (IYCF) practices among children 6-23 months of age in lowland and midland agro-ecological zones of rural Ethiopia

**DOI:** 10.11604/pamj.2016.24.163.9387

**Published:** 2016-06-27

**Authors:** Kedir Teji Roba, Thomas Pacelli O’Connor, Tefera Belachew, Nora Mary O’Brien

**Affiliations:** 1 College of Health & Medical Science, Haramaya University, Harar, Ethiopia; 2 School of Food and Nutritional Sciences, University College Cork, Cork, Ireland; 3 Department of Population & Family Health, College of Public Health & Medical Sciences, Jimma University, Jimma, Ethiopia

**Keywords:** IYCF, lowland, midland, season, dietary diversity

## Abstract

**Introduction:**

Food availability and access are strongly affected by seasonality in Ethiopia. However, there are little data on seasonal variation in Infant and Young Child Feeding (IYCF) practices and malnutrition among 6-23 months old children in different agro-ecological zones of rural Ethiopia.

**Methods:**

Socio-demographic, anthropometry and IYCF indicators were assessed in post- and pre-harvest seasons among children aged 6–23 months of age randomly selected from rural villages of lowland and midland agro-ecological zones.

**Results:**

Child stunting and underweight increased from prevalence of 39.8% and 26.9% in post-harvest to 46.0% and 31.8% in pre-harvest seasons, respectively. The biggest increase in prevalence of stunting and underweight between post- and pre-harvest seasons was noted in the midland zone. Wasting decreased from 11.6% post-harvest to 8.5% pre-harvest, with the biggest decline recorded in the lowland zone. Minimum meal frequency, minimum acceptable diet and poor dietary diversity increased considerably in pre-harvest compared to post-harvest season in the lowland zone. Feeding practices and maternal age were predictors of wasting, while women’s dietary diversity and children age was predictor of child dietary diversity in both seasons.

**Conclusion:**

There is seasonal variation in malnutrition and IYCF practices among children 6-23 months of age with more pronounced effect in midland agro-ecological zone. A major contributing factor for child malnutrition may be poor feeding practices. Health information strategies focused on both IYCF practices and dietary diversity of mothers could be a sensible approach to reduce the burden of child malnutrition in rural Ethiopia.

## Introduction

Poor Infant and Young Child Feeding (IYCF) practices are a major cause of child malnutrition. It is estimated that more than one third of child mortality in developing countries could be prevented by appropriate complementary feeding practices [1]. It is also recognized that malnutrition has short and long term adverse effects on child growth and development [2]. About one-third of deaths in children less than five years of age are due to underlying malnutrition, which includes stunting, severe wasting, deficiencies of vitamin A, zinc and iron [3]. In this study, the term malnutrition is used to refer to under nutrition. In less developed countries, 19.4% of children less than five years of age were underweight and about 29.9% were stunted in the year 2011 [4]. By 2014 prevalence of stunting in children had declined to 23.8% [5]. In 2015 in Sub Saharan Africa (SSA), 220 million people are food insecure, up to 40% of children are stunted and more than 3.4 million children less than five die each year [5, 6]. More than one-third of child deaths in Ethiopia are typically from increased severity of disease associated with malnutrition [7]. Studies in Ethiopia demonstrate that stunting is a common health problem among children less than two and ranges from 16.3 to 43% [8, 9], while wasting ranges from 1.8 to 6.8% in recently published studies [8, 10]. While many studies conducted in developing countries, including Ethiopia, indicate that the prevalence of child malnutrition is unacceptably high, few studies have investigated the seasonal effect on prevalence of child malnutrition. Studies have indicated that there is seasonal change in the prevalence of acute child malnutrition at the beginning of the dry season (October) compared to the beginning of the rainy season (May/June) [11, 12]. Similarly, high level of acute malnutrition was reported among children under 5 in Chad during the rainy season [13]. In Ethiopia it is established that food availability and access are strongly affected by seasonality; most of the households are only able to produce enough food to meet their food needs for less than six months of the year and they face food scarcities during the lean season [14, 15]. A single study conducted among children 6-36 months of age in the eastern part of Ethiopia found that the prevalence of wasting was lower in rainy (pre-harvest season) compared to dry season (7.4 vs 11.2%) [15]. Our recent study reported significant variations in nutritional status of lactating mothers in two agro-ecological zones of rural Ethiopia [16]. However, it is not known if variations in IYCF and child malnutrition follow similar patterns to that of food availability. This study reports on seasonal variation in IYCF and malnutrition among 6-23 months of age children of those lactating mothers in different agro-ecological zones of rural Ethiopia.

## Methods

### Study area

This study was conducted in the Babile, Enderta and Hintalowajirat districts of Ethiopia from January to February 2014 and July to August 2014. Babile District (Woreda), which is 560 km away from Addis Ababa in the eastern part of Ethiopia, represents lowland agro-ecological area. The altitude of Babile Woreda ranges from 950 to 2000 meters above sea level and data were collected from 1000-1500 meters above sea level. The major agricultural product for consumption is sorghum and oil seeds; and groundnuts are used as cash crop. Khat (*Catha edulis*) is also a major cash crop in this region. Hintalo Wajirat and Enderta districts (683 km and 773 km away from Addis Ababa in the northern part of Ethiopia, respectively) represent midland agro-ecological areas. Data were collected from altitudes of greater than 2000 meters above sea level where the majority produce cereals (Teff and barley) and are involved in animal husbandry. A community based longitudinal study was conducted in eight kebeles (smallest administrative unit of Ethiopia) randomly selected from each geographical area from January to February 2014 (post-harvest season which is dry) and from July to August 2014 (pre-harvest season which is rainy). Two hundred sixteen mother/child pairs were included in the study and the ages of children were 6-23 months old during post-harvest, of which 206 were surveyed again during pre-harvest season. Mothers with children 6-23 months of age were selected randomly from a registration list available in each kebele and used by researchers to verify maternal and child age. The number mother/child pairs selected in each kebele was proportional to population size in each kebele.

### Dietary data

Dietary diversity was calculated in a standardized way using a tool developed by FANTA [17, 18]. A simple questionnaire allowed all foods eaten during the 24 previous hours to be noted. Each woman involved in the study was asked to recall all the communal dishes she had eaten and given to her child in the compound during this period. Information collected allowed us to calculate a Dietary Diversity Score (DDS) using seven food groups for infants [17]: cereals/roots/tubers; pulses/nuts; vitamin A rich fruits/vegetables; other vegetables and fruits; flesh foods; eggs; milk/dairy products. A nine food group classification was used for women. Women who consumed five or more than five food groups were considered as adequate while four or greater than four food groups was considered as adequate for children. The recall was randomly made on weekdays or on weekend days, since weekends do not have any special significance with respect to dietary intake in the context of our study. We took care to not include atypical days (local feasts or celebrations) in the recall. Minimum meal frequency and minimum acceptable diet were defined and calculated according to WHO guidelines [19].

### Anthropometric data

The anthropometric measurements for mothers and children were performed using the standardized procedures recommended by WHO [19]. The study participants were weighed to the nearest 100 g on electronic scales (SECA, Germany) with a weighing capacity of 0 to 140 kg with minimal (light) clothing and removed their shoes and hats during the measurement. Children were weighed together with the mother of the child, and the child’s weight was calculated by subtracting the respective mother’s weight, and this was recorded on the form during the fieldwork and confirmed later on by supervisors. Their length/height was measured to the nearest one centimetre with locally made portable devices (SECA 2006 sliding board). The BMI was calculated by dividing weight by height in meters squared [weight/height^2^ (kg/m^2^)]. The mid-upper arm circumference (MUAC) of the left arm was measured to the nearest mm with a non-stretch measuring tape (MUAC 12.5 measuring tape/PAC-50).

### Data collectors and quality control

Data collectors were nurses holding diploma level or above qualifications. They were recruited and trained intensively on the data collection procedures, the context of specific questions across the questionnaire and anthropometric measurement procedures to be used. The questionnaire was prepared first in English then translated to Tigrigna and Afan Oromo languages as both agro-ecological zones have their own local languages. The process of data collection was overseen by supervisors and principal investigators. A pre-test survey was conducted on 5% of the total sample size in another rural area which has similar characteristics. Problems identified during the pre-test survey were corrected before the start of the actual survey. Two different measurements were taken by separate data collectors for height and weight of every study subject. In case of variation among the data collectors, the principal investigator took the measurement again for validation. Finally, the principal investigator was responsible for co-ordination and supervision of the overall data collection process. All children were apparently healthy during data collection and children with apparent sign of fever, diarrhoea, or any acute illness were excluded from the survey. Dependent variables were IYCF and nutritional status (malnutrition). The independent variables were the socio-demographic and household level characteristics of the family, health status of mothers and children, breastfeeding, housing, water and sanitation, health services utilization and cultural/social characteristics related to feeding style. Frequency of complementary feeding, dietary diversity and child illness and health seeking behaviour of the family were also assessed.

### Data processing and analysis

The data were double entered by separate data clerks into EPI Data version 3.1. Data cleaning and editing were undertaken before analyses. For analyses, data were transferred to SPSS (v 16.0) and Stata (v 11). Frequency, mean and standard deviation were computed for the variables of interest. Normality was checked graphically using different plots (P-P and/or Q-Q-plot). Assumptions including normality, homoscedasticity and linearity were checked. The WHO Anthro 2005 and ENA software of the WHO were used for calculating the Z-scores (WAZ, WLZ, LAZ, and MUACZ) and cut-off points of -2 standard deviations were used to define undernutrition. IYCF practices and women’s dietary diversity were assessed based on the UNICEF guidelines [20]. Multivariable linear regressions were applied to isolate independent effects of predictors of weight-for-height z-score, weight-for-length z-score and infant dietary diversity score and paired t-test was used to determine if significant differences existed between post- and pre-harvest seasons.

## Results

### Anthropometric status of children

Out of 216 children aged between 6 and 23 months recruited during post-harvest season, 206 (95.4%) were reassessed during pre-harvest. Reasons for loss were migration and absences from home during survey time. The main differences between pre- and post-harvest season were significantly decreased LAZ (p=0.006) and women dietary diversity score, while child weight and length were significantly increased in pre-harvest season (P<0.001). The mean infant dietary diversity score increased from 2.66 in post-harvest to 2.68 in pre-harvest season (Table 1).

**Table 1:** Descriptive statistics and paired t-test between post- and pre-harvest seasons for children aged 6-23 months in rural Ethiopia

Variables	Mean and SD		Paired t-test	
	Post-harvest	Pre harvest	Mean difference	t(p-value)
Child weight (kg)	8.21(1.4)	9.7 (4.5)	1.5 (4.3)	4.9 (<0.001)
Child length (cm)	71.4 (5.5)	77.4 (5.1)	5.8 (3.7)	22.2 (<0.001)
LAZ	-1.67 (1.4)	-1.94 (1.48)	-0.3 (1.4)	-2.8 (0.006)
WAZ	-1.30 (1.2)	-1.4 (1.2)	-0.11 (1.2)	-1.3 (0.2)
WLZ	-0.49 (1.4)	-0.52 (1.3)	-0.4 (1.6)	-0.3 (0.7)
Child MUAC (cm)	12.8 (1.4)	13.3 (4.0)	0.5 (3.9)	1.9 (0.56)
IDDS	2.66 (1.2)	2.68 (1.3)	0.01 (1.8)	0.8 (0.9)
WDDS	3.1 (0.8)	2.8 (1.6)	-0.28 (1.9)	-2.1 (0.039)

Paired t-test was conducted to identify mean change from post-harvest to pre-harvest. There is significant difference in length for age Z score, and weight and length of the children between the seasons. Post-harvest data were collected from January to February 2014 during food surplus season. Pre-harvest data were collected from July to August 2014 during food shortage season.

LAZ: Length‐for‐Age Z-score; WAZ: Weight‐for‐Age Z-score; WLZ: Weight‐for‐Length Z-score; MUAC: Mid upper arm circumferences, IDDS: Infant Dietary Diversity score; WDDS: Women dietary diversity score; SD: Standard deviation.

### Prevalence of child malnutrition

For the children in this study 11.6% were wasted, 26.9% were underweight, and 39.8% were stunted in post-harvest season. In pre-harvest (lean) season, the prevalence changed to 8.5%, 31.8% and 46.0%, respectively (Table 2). Regarding the change in prevalence across agro-ecological zones, a greater change in stunting was recorded in the midland agro-ecological zone (from 36.2% post-harvest to 46.9% pre-harvest) compared to lowland (from 43.2% post-harvest to 45.3% pre-harvest) (Table 3). On the other hand, the prevalence of acute malnutrition (wasting) declined by half in lowland region (from 12.6% post-harvest to 6.6% pre-harvest) with no seasonal change in their midland counterparts (Table 3).

**Table 2:** Seasonal variation in malnutrition and Infant and Young Child Feeding (IYCF) practices among children 6-23 months of age in rural Ethiopia

Variables	Post-Harvest		Pre- Harvest		X^2^ (P-Value)
	No	%	No	%	
Wasted	25	11.6	17	8.5	5.4(0.020)
Underweight	58	26.9	64	31.8	15.1(<0.001)
Stunted	86	39.8	93	46.0	48.9(<0.001)
Received minimum meal frequency	109	50.5	107	51.9	0.26(0.61)
Infant Dietary Diversity (≥ 4 food groups)	48	22.2	52	24.1	5.9(0.015)
Received minimum acceptable diet	26	12.0	34	16.6	1.95(0.16)
Women dietary diversity (≥ 5 food groups)	13	6.0	32	16.7	0.48(0.001)
Currently breastfed	200	92.6	184	89.3	0.32(0.57)
Bottle fed yesterday	130	60.2	86	39.8	13(0.0003)
Group 1, grains, roots and tubers	187	86.6	206	95.4	1.6(0.20)
Group 2, legumes	120	55.6	139	64.4	0.049(0.89)
Group 3, dairy products	102	47.2	51	23.6	20.3(<0.001)
Group 4, flesh food	4	1.9	16	7.4	1.8(0.18)
Group 5, egg	11	5.1	8	3.7	0.44(0.50)
Group 6, pre-vitamin A rich fruit and veg	10	4.6	70	32.4	3.64(0.56)
Group 7, other fruits and vegetables	133	61.6	95	44.0	2.3(0.13)

Higher level of stunting and wasting in pre-harvest season compared to post-harvest, but low level of underweighted in pre-harvest season. Higher consumption of fruit and vegetable, and dairy were reported in post-harvest season. Post-harvest season is food surplus season and pre-harvest is food shortage season

**Table 3:** Malnutrition and Infant and Young Child Feeding (IYCF) practices among children 6-23 months of age by agro-ecology and sex in rural Ethiopia

Seasons	Category	Agro-ecology		OR (95%CI)	Child Sex		OR (95%CI)
		Lowland^1^ N (%)	Midland^2^ N (%)		Male N (%)	Female N (%)	
**Post-harvest**	Wasted	14(12.6)	11(10.5)	0.73(0.28,1.85)	13(13.5)	12(10.0)	1.4(0.6,3.25)
	Underweight	32(28.8)	26(24.8)	0.65(0.25,1.65)	28(29.2)	30(25.0)	1.2(0.67,2.26)
	Stunted	48(43.2)	38(36.2)	0.74(0.41,1.33)	46(47.9)	40(33.3)^*^	1.8(1.1,3.2)*
	Received minimum meal frequency	38(34.2)	71(67.6)	4.01(2.2,7.36)*	46(47.9)	63(52.5)	1.2(0.7,2.06)
	IDDS (≥ 4 food groups )	31(27.9)	17(16.2)	0.49(0.24,1.01)	19(19.8)	29(24.2)	1.3(0.67,2.5)
	Received minimum acceptable diet	14(12.6)	12(11.4)	0.9(0.36,2.23)	10(10.4)	16(13.3)	1.0(0.8,1.3)
	WDDS (≥ 5 food groups)	12(10.8)	1(1.0)	0.07(0.001,0.55)*	____	____	____
	Child drank milk in previous 24 h	78(70.3)	24(22.9)	0.12(0.06,0.24)*	49(51.0)	53(44.2)	0.75(0.42,1.3)
	Child ate food rich in iron	1(0.9)	3(2.9)	3.2(0.25,171.3)	3(3.1)	1(0.8)	0.26(0.005,3.3)
**Pre-harvest**	Wasted	7(6.6)	10(10.5)	0.6(0.22,1.65)	9(10.2)	8(7.1)	1.5(0.55,4.05)
	Underweight	30(28.3)	34(35.8)	0.71(.39,1.3)	30(34.1)	34(30.1)	1.2(0.66,2.2)
	Stunted	48(45.3)	45(46.9)	0.94(.54,1.63)	46(51.7)	47(46.0)	1.5(0.86,2.6)
	Received minimum meal frequency	66(59.9)	41(39.6)	0.45(0.24,0.82)*	37(42.0)	70(61.9)	5.0(2.6,9.6)*
	IDDS (≥ 4 food groups )	49(44.5)	3(2.9)	0.049(0.01,0.17)*	25(26.3)	27(22.5)	1.0(0.5,1.9)
	Received minimum acceptable diet	31(28.2)	3(2.9)	0.078(0.015,0.27)*	14(15.9)	31(27.4)	3.2(1.5,7.0)*
	WDDS(≥ 5 food groups)	32(36.8)	0(0.0)	___	___	___	____
	Child drank milk in previous 24 h	51(46.4)	0(0.00)	___	27(28.4)	24(20.0)	0.63(0.33,1.18)
	Child ate food rich in iron	12(10.8)	4(3.8)	0.34(0.08,1.2)	8(8.3)	8(6.7)	1.1(0.34,3.6)

There are significant variations across agro-ecological zones in WDDS, drinking milk in 24 hr, minimum meal frequency, IDDS and minimum acceptable diet. There is significantly higher rate of stunting among male than female in post-harvest season, and higher level of receiving minimum meal frequency and minimum acceptable diet in among female in pre-harvest season.

IDDS: infant dietary diversity calculated from seven food group, WDDS:Women dietary diversity score calculated from nine food group.

1 Babile District (Woreda) which is 560 km away from Addis Ababa in the eastern part of Ethiopia is representing a lowland agro-ecological zone.

2 Hintalo Wajirat and Endreta districts are 683 km and 773 km away from Addis Ababa in the northern part of Ethiopia, respectively and represent midland agro-ecological zones.

### Child feeding practices

Grains, roots and tubers were the most common food items consumed by the children in the previous 24 hours in both seasons, followed by legumes and other fruit and vegetables. It was found that consumption of grains, legumes and pre-vitamin A rich fruit and vegetables increased in pre-harvest season, while consumption of egg, milk and other fruits and vegetables declined in pre-harvest compared to post-harvest season (Table 2). The majority of mothers were breastfeeding their children beyond one year of age as 89.3% of the mothers still breastfed their child during the second round of the survey (lean season) (Table 2).

### Dietary diversity

The proportion of children overall who received minimum dietary diversity (≥ 4 food groups) was 22.2% in post-harvest and increased to 24.1% in the pre-harvest season (Table 2). Similarly, the proportion of children overall who received minimum meal frequency and minimum acceptable diet were slightly increased in the pre-harvest season (Table 2). However, when one examines the data by agro-ecological zones, the proportion of children who received minimum dietary diversity in the midland region declined from 16.2% post-harvest to 2.9% pre-harvest while it significantly increased in the lowland region from 27.9% post-harvest to 44.5% pre-harvest (Table 3). This variation between agro-ecological zones was significant (P<0.05) (Table 3). The percentages of children who received minimum meal frequency and minimum acceptable diet were significantly higher in the lowland zone compared to midland in the pre-harvest season and female children had higher percentages than male (Table 3). There was a slightly higher rate of wasting, underweight and stunting among male compared to female children in both seasons. There was a statistically significant difference in stunting between male and female children during the post-harvest season (p<0.05). Likewise, there were slightly higher rates in measures of IYCF practices among female compared to male children with the exception of drinking milk, where the rate was higher among male children (Table 3).

### Predictors of malnutrition and dietary diversity

Maternal age, minimum meal frequency, height of the mother and age of the children were predictors of child weight for length Z-score (WLZ) in post-harvest season. Children who received minimum meal frequency were less likely to be wasted compared to counterparts (p=0.092). As the age of the mothers increased by one year, the risk of the child WLZ decreased by 0.047(β=-0.047, P=0.001). Similarly, as the height of the mothers increased by one centimetre, the risk of the child being wasted decreased (β=-0.048, P=0.002). Those children aged greater than 12 months had lower WLZ compared to children aged less than 12 months of age (β=-0.057, P=0.002). In model four, age of mother and infant dietary diversity score were independent predictors of WLZ in pre-harvest season (Table 4). It was found that infant feeding practice is a significant predictor of wasting in both seasons (Table 4). In model two, infant dietary diversity score in the post-harvest season was significantly associated with women dietary diversity score and child age. As dietary diversity of women increased by one unit, the dietary diversity of the child also increased (β=0.2 P=0.042). Similarly, as the age of the child increased by one month, the dietary diversity score of the children increased by 0.84 units (β=0.84, P<0.001). In model five, women’s dietary diversity is a predictor of infant dietary diversity score. In general, women dietary diversity score is an independent predictor of infant dietary diversity score in both seasons, while agro-ecological region does not show significant association with child dietary diversity in multivariable analysis (p=0.27) (Table 4).

**Table 4:** Selected predictors of malnutrition and dietary diversity among children 6-23 months of age in rural Ethiopia, multivariate regression

Variables	β(95% CI)	P values
**Model 1: Post-harvest WLZ**		
Child received minimum meal frequency	-0.029(-0.63,-0.05)	0.092
Age of mothers (years)	-0.047(-0.07, -0.019)	0.001
Height of mothers	-0.048(-0.08,-0.019)	0.002
Age of child as greater or less than 12 months	-0.057(-0.93,-0.023)	0.002
**Model 2: Post-harvest IDDS**		
Women dietary diversity score (WDDS)	0.2(0.01 0.37)	0.042
Age of the child (months)	0.84(0.52, 1.2)	<0.001
**Model 3: Pre-harvest LAZ**		
Child MUACZ score	0.37(0.21,0.52)	<0.001
Age of the child (months)	-0.042(-0.08,0.001)	0.47
IDDS	0.13(-0.022,0.28)	0.093
Region	0.13(-0.28,0.54)	0.5
**Model 4: Pre-harvest WLZ**		
Age of mothers (years)	-0.033(-0.06,-0.01)	0.02
Child MUACZ score	0.36(0.22,0.49)	<0.001
Region	-0.067(-0.44,0.31)	0.71
IDDS	0.42(-0.03,0.86)	0.068
**Model 5: Pre-harvest IDDS**		
WDDS	0.44(0.33,0.55)	<0.001
Age of mothers (years)	0.023(-0.004, 0.05)	0.092
Region	0.21(-0.16,0.58)	0.27

Age and height of the mother are predictors of WlZ in post-harvest season while only age of the mother is significant in pre-harvest season. WDDS is significantly associated with IDDS in both seasons. As MUAC of the children increase the LAZ of the children also significantly increase. LAZ: Length-for-Age Z-score, β, coefficient for the predictor value; MUACZ: Mid Upper Arm Circumference for Age Z-score; WLZ: Weight‐for‐Length Z-score. IDDS: Infant Dietary Diversity Score; WDDS: Women Dietary Diversity Score

## Discussion

Investigation of effects of seasonal variation on nutritional status of children is more complicated than for lactating mothers who, as adults, have stopped growing. This is because groups of children living in underprivileged environments consistently show an evolution of malnutrition over time, even in the total absence of seasonality effects. In resource poor settings, children after six months of age tend to show a marked decline in nutritional status due to changes in diet and morbidity and this deterioration probably occurs regardless of season. The post-harvest and pre-harvest sampling periods in this study were separated by an interval of six months, which is long enough for age-related dynamic effects in children to manifest themselves. Since the same children were assessed at both time points, and all of these children naturally grew between the data collection periods, interpretation of the data must take care not to attribute all changes solely to seasonality. In this study we found that 39.8% of children were stunted, 11.6% wasted and 26.9% were underweight in the post-harvest season while these prevalences were changed to 46.0%, 8.5% and 31.8%, respectively, in the pre-harvest season. These findings highlight the magnitude of malnutrition as a major public health problem among children aged 6-23 months of age in rural Ethiopia. Prevalence of stunting increased but wasting declined from post- to pre-harvest seasons. Malnutrition can start very early in life and progressively increase during the child’s growth. The major change in stunting from post- to pre-harvest seasons was seen in the midland compared to the lowland region (increased from 36.2% to 46.9% in midland, and from 43.2% to 45.3% in lowland). The proportion of children who were wasted was decreased by nearly half from 12.6% post-harvest to 6.6% pre-harvest in lowland region. There was no change in the prevalence of wasting between seasons in children from the midland region. The reasons for the variation between agro-ecological zones with respect to stunting and wasting were not clear. The proportion of children who had adequate dietary diversity was 2.9% in midland zone in the lean (pre-harvest) season whereas 44.5% of children in lowland received adequate dietary diversity in the lean season. In the midland region it was noted that most mothers participated in weeding of cereals and this may lead to less time available to care for their children, while in the lowland region, Khat production requires less weeding than cereal crops and, additionally, Khat production was primarily handled by males (data not shown).

The community in the lowland zone relies primarily on cash crop production (Khat, *Catha edulis*) and this activity increased during the rainy season (pre-harvest). This product is sold to market and monies obtained may be used to purchase foodstuff and hence help to bridge the gap in food availability between seasons. This community also produce vegetables and fruit which, to some extent, are consumed at household level. The same seasonal changes in rates of acute under nutrition were reported in a previous study from a similar setting of eastern Ethiopia [15]. Additionally, our investigations among mothers of children in this study also found that women from the midland region were more affected by lean season (pre-harvest) compared to their counterparts in the lowland region [16]. Stunting is an indicator of chronic malnutrition and is a hindrance to linear growth, which is the product of a cumulative history of stressful episodes that may be compensated by catch-up growth during more favourable periods. The prevalence of stunting in our study was greater than reported in some other studies [8, 21, 22], and lower than the EDHS in post-harvest season but higher than EDHS in pre-harvest season [23]. These variations in stunting could be attributed to different socio-demographic and economic situations in the study areas. We found that infant dietary diversity score is weakly associated with child stunting in the pre-harvest season, indicating there is a possible association between stunting and feeding practice unlike another study reported from Ethiopia [9]. Similarly, the overall proportion of children underweight was increased in pre- compared to post-harvest season with higher change in the midland agro-ecological zone and no significant change in the lowland zone. The proportion of underweight children in our study is larger than reported in other recent studies [8, 10, 22], and similar to EDHS in post-harvest season [23]. The main contributor to the rise in underweight children in the midland agro-ecological zone may be partly attributed to poor feeding practices during the lean season compared to the post-harvest season.

We found that shorter stature mothers are more likely to have wasted children, which suggests that malnutrition is intergenerational from mother to child, a finding that is also supported by other studies [24]. Similarly, children from mothers with lower dietary diversity score are more likely to have lower dietary diversity score themselves persistently in both seasons. These data indicate that children born in households with inadequate feeding practices tend to have poor dietary practices also. This may be associated with household food insecurity or poor knowledge about the importance of household dietary diversity. The association between maternal and infant dietary scores was also identified in studies in Bangladesh, Vietnam, and elsewhere in Ethiopia, which indicated that, as the dietary diversity of the mother’s increases by one unit, the dietary diversity of infant’s increases by 0.24 dietary groups [25]. Therefore, interventions that focus on infant feeding practices should also consider in parallel maternal nutrition and dietary diversity practices. This study also revealed that low weight-for-length Z-score (WLZ) in children was associated with low dietary diversity and low minimum meal frequency. This indicates that poor feeding practices are a key cause of wasting in both seasons which needs urgent intervention. Likewise, as the age of the mothers increases, the risk of the child being wasted decreases. Nearly all mothers in Ethiopia breastfeed their children. However, the proportion exclusively breastfeeding before 6 months and providing appropriate complementary feeding thereafter was very low compared to WHO recommendations [26]. This study found that the vast majority of the mothers were breastfeeding their children and about 89.3% of them continued breastfeeding beyond one year of age, which is similar to findings in a study conducted by Regassa, who reported that 87% of mothers continued breastfeeding beyond the first year in southern Ethiopia [27]. It is accepted that continued and frequent breastfeeding is important for a child’s health and decreases the risk of morbidity and mortality in underprivileged populations [28]. Despite the fact that a large proportion of mothers had long duration of breastfeeding, complementary feeding practices are poor in our study areas. As indicated above, the proportion of children who receive appropriate complementary feeding was very low. Studies in Ethiopia showed that even mothers who had knowledge on the timing and importance of complementary feeding do not give appropriate complementary feeding to their children [10]. This may be associated with cultural and traditional backgrounds of the family.

The 24 hour dietary diversity score measured in this survey indicated that most of the children do not achieve appropriate dietary diversity as recommended by WHO [26], as less than 25% of children received four or greater food groups in the 24 hours preceding the survey in both seasons. This finding is similar to previous studies indicating that children in this age category received less than four food groups. The exceptions are studies conducted by Negash et *al*. [10] and Regassa [27] who reported that 32.5% and 42.4%, respectively, of children received ≥ four food groups. Regarding seasonal variation, there was a slight increase in pre- compared to post-harvest season in dietary diversity. Our findings indicate that there was disproportional decline in the measures of IYCF practices such as dietary diversity, minimum meal frequency and minimum acceptable diet in midland agro-ecological zones between post- and pre-harvest seasons while these indicators increased in the lowland zone over the same period. Thus, children in the midland zone were more affected by the lean season than their counterparts in the lowland zone. The most commonly consumed food groups by children were grains, roots and tubers, followed by legumes, and other fruit and vegetables in both seasons with slight decrease in other fruit and vegetables in the lean season. Higher consumption of grains, roots and tubers and other fruit and vegetables are typical characteristics of the Ethiopian diet [8, 22]. But higher rate of legume consumption is more typical of the northern Ethiopian regions [29]. Consumption of milk is significantly decreased during lean season in both agro-ecological zones. However, our data indicate that all children did drink milk in the midland region in the 24 hours preceding the survey. The consumption of milk in these study areas is higher than reported in others studies [8, 29] and lower than reported by Regessa and Mesfin et al. [22, 27]. Similarly, consumption of meat and egg were low in this study as most communities in rural parts of Ethiopia do not consume meat regularly [8]. Consumption of meat and egg by our study participants was lower than studies in similar setting [22, 29]. The strong point of this study is its design (longitudinal study design). We were able to investigate IYCF practices and anthropometric indices in this transition period from breastfeeding/predominately breastfeeding to complementary food. This age group is at high risk for many infectious diseases, and inappropriate feeding practices particularly in a developing country, as well as being in the window period where the effects of malnutrition are not adequately averted at later age of development.

There are limitations in our study that might affect our findings. Most of the risk factors were determined from maternal reports as there was no other means of obtaining that information for the children, this may introduce recall bias. Nonetheless, the duration prior to recall was short and we included some double checking in the questionnaire for validation of results. Thus, maternal recall bias is unlikely to have affected the observed relationships.

## Conclusion

In conclusion, this study showed that malnutrition and poor IYCF practices among children aged 6-23 months of age are major public health concerns in rural Ethiopia. Poor IYCF practices among children in both seasons may be the major underlining causes of malnutrition. Results suggest that the lean season affects children of the midland agro-ecological zone more than the lowland region. However, children in the midland zone had better anthropometric measures and IYCF practices in the post-harvest season but these indices deteriorated significantly during the lean season compared to the lowland children. On the other hand, the indices of malnutrition and IYCF practices among the lowland children were very poor in both seasons. Interventions focusing on improving appropriate IYCF practices at an early age should be in place to prevent malnutrition and its devastating impact with special focus on agro-ecological zones.

### What is known about this topic

- Under nutrition is a common health problem in Ethiopia;
- Breast feeding is universal but there are poor Child Feeding practices in Ethiopia.

### What this study adds

- There is seasonal and agro-ecological variation in under nutrition among children 6-23 months of age;
- Seasonal and agro-ecological variation in feeding practices among children 6-23 months of age has not been previously reported in rural Ethiopia.
